# Facile Synthesis of Dual-Network Polymer Hydrogels with Anti-Freezing, Highly Conductive, and Self-Healing Properties

**DOI:** 10.3390/ma17061275

**Published:** 2024-03-10

**Authors:** Yuchen Jin, Lizhu Zhao, Ya Jiang, Xiaoyuan Zhang, Zhiqiang Su

**Affiliations:** State Key Laboratory of Chemical Resource Engineering, Beijing Key Laboratory of Advanced Functional Polymer Composites, Beijing University of Chemical Technology, Beijing 100029, China; 2020020454@buct.edu.cn (Y.J.); 202002046603@buct.edu.cn (L.Z.); 2021200427@buct.edu.cn (Y.J.)

**Keywords:** dual-network hydrogels, swelling resistance, frost resistance, conductivity, self-healing

## Abstract

We report the synthesis of poly(acrylamide-co-acrylic acid)/sodium carboxy methyl cellulose (PAMAA/CMC-Na) hydrogels, and subsequent fabrication of dual-network polymer hydrogels (PAMAA/CMC-Na/Fe) using as-prepared via the salt solution (FeCl_3_) immersion method. The created dual-network polymer hydrogels exhibit anti-swelling properties, frost resistance, high conductivity, and good mechanical performance. The hydrogel swells sightly when immersed in solution (pH = 2~11). With the increase in n_AA_:n_AM_, the modulus of elasticity experiences a rise from 1.1 to 1.6 MPa, while the toughness undergoes an increase from 0.18 to 0.24 MJ/m^3^. Furthermore, the presence of a high concentration of CMC-Na also contributes to the enhancement of mechanical strength in the resulting hydrogels, ascribing to enhanced physical network of the hydrogels. The minimum freezing point reaches −21.8 °C when the CMC-Na concentration is 2.5%, owing to the dissipated hydrogen bonds by the coordination of Fe^3+^ with carboxyl (-COO^−^) in CMC-Na and PAMAA. It is found that the conductivity of the PAMAA/CMC-Na/Fe hydrogels gradually decreased from 2.62 to 0.6 S/m as the concentration of CMC-Na rises. The obtained results indicates that the dual-network hydrogels with high mechanical properties, anti-swelling properties, frost resistance, and electrical conductivity can be a competitive substance used in the production of bendable sensors and biosensors.

## 1. Introduction

Hydrogels exhibit promising potential for utilization in flexible wearable sensors [1], tissue engineering [2], biomedicine [3], and intelligent robots [4], as a result of their high water content [5], good viscoelasticity [6], well transparency [7], and high biocompatibility [8]. Hydrogel-based electronic devices have garnered growing interest as a result of their remarkable flexibility and extensibility [9]. Previously, a lot of functional hydrogels have been prepared from gelatin [10], chitosan [11], sodium alginate [12], and other biomass through physical cross-linking, such as electrostatic interaction and hydrogen bonding. Alternatively, hydrogels can be produced through the process of chemical cross-linking using covalent interactions, for example, acrylamide (AM) and acrylic acid (AA) [13].

Swelling resistance is a desirable function in hydrogel applications [14]. Most hydrogels contain many hydrophilic functional like hydroxyl (-OH), amino (-NH_2_), and acylamino (-CO-NH_2_) in the main chain structure, resulting in the hydrogel absorbing external water molecules and volume expansion in the humid environment (RH > 75%) [15]. Thus, in a liquid environment, hydrogels will inevitably expand in volume [16]. However, with the increase in the swelling duration, the pore wall of the hydrogel becomes irreversibly thinner [17]. The network structure loses its rigidity, leading to the fragmentation and the decrease in the strength and toughness of hydrogels. This limitation jeopardizes their potential applications across various fields [18]. Li et al. prepared poly(acrylic acid-*co*-N,N’-dimethylacrylamide)/aluminum hydroxide (P(AA-*co*-DMAA)) nanoparticle hydrogels with good swelling-resistance [19]. The formation of this hydrogel can be attributed to the effective coordination between carboxyl groups and Al(OH)_3_ nanoparticles from P(AA-*co*-DMAA). Therefore, the designed hydrogel could stably exist in seawater with negligible swelling.

A dual-network hydrogel is composed of a rigid and fragile network structure containing bonds that dissipate energy, along with a flexible and stretchable network structure featuring extensible bonds. It possesses not only high mechanical strength but also anti-swelling performance [20]. When the dual-network hydrogel is subjected to sufficient load, the rigid and brittle networks are destroyed first, dissipating external energy. The protection of the soft and ductile network enhances the hydrogel properties both in mechanical strength and toughness, preventing any potential damage [21]. Meanwhile, the soft and tough network provides supports for mechanical strength, keeping its original shape undamaged [22]. The network structures penetrate each other inside the hydrogel and improve the cross-linking density of the hydrogel network structure. Furthermore, the formation of dual-network in hydrogel increases the porosity between the internal networks of the hydrogel, so as to enhance the anti-swelling capability of the dual-network hydrogel to surpass that of the single-network hydrogel [23]. It is reported that the crosslinking of hydrogen bonds in one-pot synthesis may produce free radicals in CMC-Na, which form covalent bonds with monomers in polymerization, forming a hybrid network without FeCl_3_. Therefore, different processes must be used in order to ensure the crosslinking function of Fe^3+^. However, it is evident that ion crosslinking increasing the rigid cross section in the polymer network, which enhanced the anti-swelling properties of the hydrogel [24].

Refraining from maintaining their elasticity and conductivity at low temperatures, conventional conductive hydrogels encounter limitations in their applicability under specific circumstances [25]. Most of the existing studies have concentrated on enhancing the characteristics related to mechanics and sensing capabilities in hydrogels. [26]. However, only a few studies have discussed how to overcome their functional attenuation when it comes to extreme conditions, for instance the hypoxia, low temperature, and high-dose ultraviolet radiation. For instance, Morelle et al. synthesized the polyacrylamide-alginate dual-network hydrogels [27]. The hydrogels were then soaked into CaCl_2_ solutions to decline their freezing point and they exhibited a relatively high toughness. The hydrogels that formed in 10 wt% and 30 wt% CaCl_2_ maintained their stretchability at −30 and −50 °C relatively.

Hydrogels can be classified into two categories: electron conducting hydrogels and ion conducting hydrogels. Electron conducting hydrogels facilitate charge transfer through the migration of electrons or holes in conductive polymers, which are further categorized as p-type and n-type. On the other hand, ionic conductive hydrogels do not require additional conductive fillers; their conductivity is achieved through the migration of uniformly distributed conductive ions within the hydrogel framework, resulting in stable electrochemical properties. Sufficient ionic conductivity is essential for hydrogels to reduce the loss of voltage between electrolyte resistance and electrode to avoid low discharge rate [28]. Ji et al. immersed a polymer hydrogel into 1 M Li^+^ solution to hold sufficient cations [29]. The hydrogel achieved a significant ion conductivity level of 2.2 mS/cm, which could be a suitable material for the solid gel electrolyte membrane of energy storage devices. Liu et al. reported the synthesis of cross-linked PVA/PA-Fe hydrogels via one-pot method supported by phytic acid (PA) and ferric trichloride [30]. The existence of both Fe^3+^ and Cl^−^ ions originating from FeCl_3_ endowed the created hydrogels with tunable ionic conductivity.

**Figure 1:**
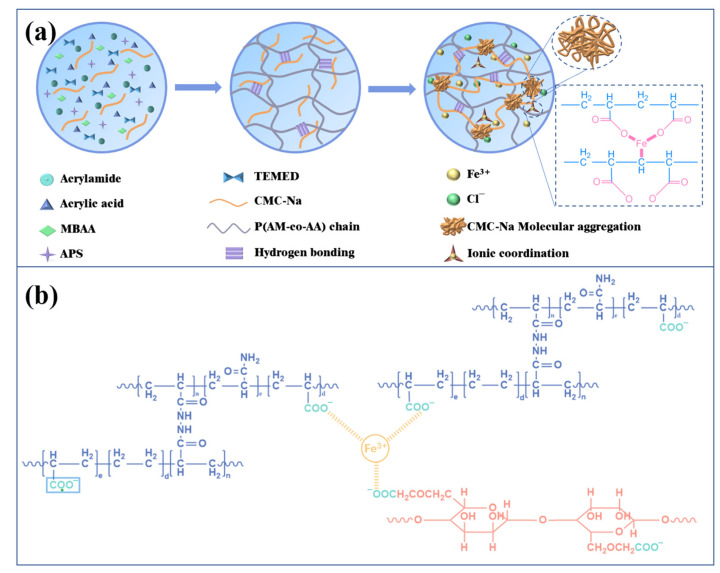
(**a**) Schematic representation depicting the synthesis procedure of PAMAA/CMC-Na/Fe hydrogels. (**b**) Cross-linking of -COO^−^ with Fe^3+^ in the hybrid hydrogels.

In this study, we prepare multifunctional hybrid dual-network polymer hydrogels by immersing the as-prepared hydrogels into FeCl_3_ solution to achieve both high swelling resistance and high conductivity. As shown in Figure 1a, the poly(acrylamide-*co*-acrylic acid)/sodium carboxy methyl cellulose (PAMAA/CMC-Na) hydrogels are firstly synthesized by one-pot method, which are then treated with FeCl_3_ to create dual-network PAMAA/CMC-Na/Fe hydrogels. In the polymerized structure of the hydrogels, the covalent cross-linking between AM and AA in PAMAA formed a chemical network through chemical bonding. Subsequently, CMC-Na and poly (AM-*co*-AA-*co*-MBAA) form a double network by crosslinking ions with Fe^3+^ and as indicated in Figure 1b. The interaction and bonding between Fe^3+^ and -COO^−^ in hydrogels not only impart remarkable electrical conductivity and mechanical properties to the hydrogels, but also bolster their resistance against swelling. The conductivity of PAAMA/CMC-Na/Fe hydrogels is attributed to free ions Fe^3+^ and Cl^−^ present within these gels.

## 2. Experimental Section

### 2.1. Materials and Chemicals

AM (98% in purity), AA (98% in purity), CMC-Na (1000 cps), *N,N*’-methylene-bisacrylamide (MBAA, 99% in purity), ammonium persulfate (APS, 98% in purity), *N,N,N*’,*N*’-tetramethyl-ethylenediamine (TEMED, 99% in purity), and FeCl_3_·6H_2_O (97% in purity) were bought from the J&K Chemical Ltd(Shanghai, China). DI water (18.25 MΩ) was employed throughout the experiments.

### 2.2. Synthesis of Dual-Network PAMAA/CMC-Na/Fe Hydrogels

The PAMAA/CMC-Na/Fe hydrogels were synthesized using a two-step strategy, which involved free radical polymerization followed by immersion. To prepare a homogeneous and transparent solution, deionized water (10 mL) was used as the solvent. AM (2.2 g), AA (1:20, 1:10, 3:20, 1:5, and 1:4 molar ratios of AA/AM), and CMC-Na (0.5, 1.0 1.5, 2.0 and 2.5 wt%) were added to the water mixture. Molar ratios of AA/AM is defined as n_AA_:n_AM_. The resulting solution consisted of CMC-Na, polymer monomers, cross-linker (MBAA), APS, and TEMED. The mixed solution was dispersed with a magnetic stirrer at a temperature of 25 °C for a duration of 30 min ensured proper mixing. Subsequently, MBAA (0.002 g), APS (0.3 g, 1 wt%), and TEMED (0.4 g, 2 wt%) were added into the acquired clarified solution. After adding and mixing the drug, the sample is transferred onto a surface dish with a height of approximately 2 mm and undergoes hot polymerization at 60 °C for six hours. The resulting hydrogel was synthesized through thermo-polymerization of this mixture. This synthesis process led to the formation of a cross-linked chemical network consisting of PAMAA/CMC-Na hydrogel structure. With the hydrogen bond between CMC-Na chains and the covalent network of PAMAA, an even distribution of the CMC-Na chains within the PAMAA network was achieved. In the second step, the as-prepared PAMAA/CMC-Na hydrogel was immersed in an aqueous solution containing FeCl_3_ (0.02, 0.04, 0.06, 0.08, and 0.10 M) for a duration of 3 h to form dual-network PAMAA/CMC-Na/Fe hydrogel. The volume ratio between the sample and the Fe^3+^ solution is approximately 1:1. The PAMAA/CMC-Na/Fe/NaCl hydrogel was prepared by incorporating 1.8 wt% NaCl into the CMC-Na solution, followed by mixing with other drugs and subjecting to hot polymerization at 60 °C in an oven. An inert atmosphere was not used in the synthesis procedure. The specific compositions for all the prepared hydrogels are recorded in Table 1.

### 2.3. Water Contents of Hydrogels

The hydrogels were measured on a balance and the weight was noted as *W*_wet_. Subsequently, the hydrogels were subjected to drying in an oven at a temperature of 110 °C until their weight reached a stable state. The final weight was recorded as *W*_dry_. Relevant to the weight reduction of hydrogels, it was determined that the decrease in mass could be primarily attributed to the presence of water. The quantification of water content was calculated as (*W*_wet_ − *W*_dry_)/*W*_dry_. The mean values were derived by considering a minimum of three data points for every hydrogel.

### 2.4. Measurement of Anti-Swelling Properties of Hydrogels

The swelling properties of PAMAA/CMC-Na/Fe dual network hydrogels soaked in different pH conditions were investigated. The hydrogels were measured using a balance and the initial weight was recorded as W_0_. Then, they were soaked in solutions (pH = 1~14). The weight after swelling each day was noted as *W*_sw_. The swelling ratio was determined as *W*_sw_/*W*_0_, while the weight at equilibrium was documented as *W*_eq_. Afterwards, the hydrogels underwent drying in an oven set at 110 °C until their weight stabilized. The final weight was recorded as *W*_dry_, and the equilibrium water content was calculated as (*W*_eq_ − *W*_dry_)/*W*_eq_. To ensure accuracy, average values were obtained from no less than three data points for each hydrogel sample.

### 2.5. Characterization of Swelling-Resistant Properties under Different pH

The swelling resistance of hydrogels was measured by equilibrium water content and swelling ratio. The hydrogels were measured using a balance and the initial weight was recorded as *W*_0_. Subsequently, they were immersed in various solutions with pH values ranging from 1 to 14. HCl and NaOH were used to obtain a solution with a pH of 1–14, and the anti-swelling test was performed at different pH values. The weight of the hydrogels after swelling each day was noted as *W*_sw_, and the swelling ratio was determined as *W*_sw_/*W*_0_. The weight at equilibrium was documented s as *W*_eq_. Following this, the hydrogels were subjected to drying in an oven set at 110 °C to achieve a constant weight which was then recorded as *W*_dry_. The equilibrium water content was calculated by (*W*_eq_ − *W*_dry_)/*W*_eq_. To ensure accuracy, a minimum of three data points for each hydrogel were considered when deriving mean values. The drying environment for hydrogels is maintained around or below 110 °C, ensuring only water within the hydrogels is consumed.

### 2.6. Anti-Freezing Properties of Hydrogels

Differential scanning calorimeter (DSC) measurements of the hydrogels were performed on a Q200 DSC (TA Instruments, Delaware State, America) under 50 mL/min nitrogen flow. The hydrogels were firstly kept at 25 °C and then the anti-freezing tests were carried out by cooling the hydrogels to −50 °C with a cooling rate of 5 °C/min.

### 2.7. Mechanical Properties of Hydrogels

The hydrogels were evaluated using an MTS E44.104 testing machine equipped with a 250 N load cell at ambient temperature. Each hydrogel was subjected to three tests. To measure tensile properties, it was essential that the hydrogels were shaped into dumbbell samples following the guidelines of GB/T-528-2009 standard [31] (4 mm inner width, 10 mm gauge length, and 1–2 mm thickness). The extension rate during testing was set at 50 mm/min. For cyclic tensile testing, dumbbell-shaped samples with a 4 mm inner width and a 30 mm gauge length were prepared, and the loading–unloading speed was maintained at 100 mm/min.

The tensile stress was calculated by dividing the applied force by the area of the cross-section, expressed as σ = *F*/*A*_0_. The formula ε = (*l* − *l*_0_)/*l*_0_, where l represents the final length and *l*_0_ denotes the initial length of the sample, was used to calculate the tensile strain. To calculate the elastic modulus (E), we analyzed the slope within a 5–15% range on the stress–strain curve. Additionally, we quantified toughness by measuring the area enclosed between the stress–strain curve and strain axis.

### 2.8. Conductivity of Hydrogels

The effects of different concentrations of CMC-Na, n_AA_/n_AM_, Fe^3+^ concentration, and soaking time in the Fe^3+^ solution on the conductivity of PAMAA/CMC-Na/Fe dual network hydrogels were investigated.

The previously prepared hydrogel was precisely sectioned into a cylindrical sample with a radius of 6 mm and an identical thickness of 2 mm. The stable voltage was tested using an electrochemical workstation CHI760E(CH Instruments, Inc., Shanghai, China) in Open Circuit Potential Time mode. The corresponding resistance was tested in A.C. Impedance mode. The formula involved σ = *L*/*RS*, where σ represents the conductivity, R represents resistance and S represents cross sectional area, was used to calculate the conductivity. Moreover, the samples tested for electrical conductivity were not treated with swelling, but were tested with the original hydrogel.

## 3. Results and Discussion

### 3.1. Water Content of PAMAA/CMC-Na/Fe Hydrogels

**Figure 2:**
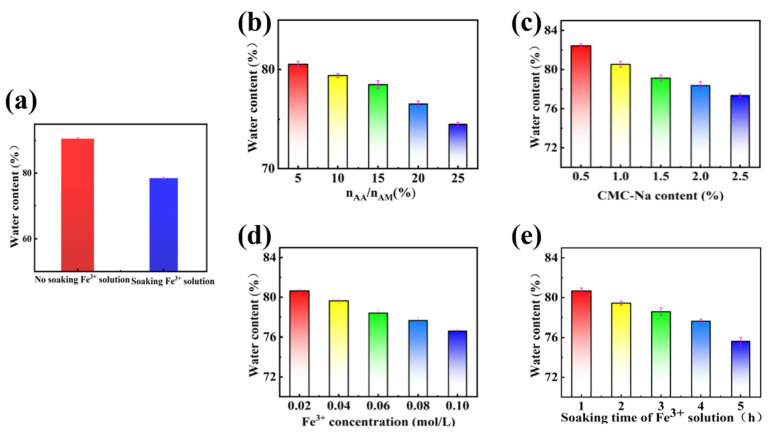
(**a**) Water content of PAMAA/CMC-Na and soaking PAMAA/CMC-Na/Fe; Effects of different (**b**) n_AA_:n_AM_, (**c**) CMC-Na concentration, (**d**) Fe^3+^ concentration, and (**e**) different soaking time on water content.

The prepared hydrogels of PAMAA/CMC-Na and PAMAA/CMC-Na/Fe were subjected to a heat treatment at 110 °C for 24 h in an oven. Then their water content was determined. As depicted in Figure 2a, the water content of PAMAA/CMC-Na hydrogel (90.57%) was significantly higher than that of PAMAA/CMC-Na/Fe hydrogel (78.45%). The main chain structure of PAMAA/CMC-Na hydrogel contains a large number of hydrophilic groups such as hydroxyl (-OH), amino (-NH_2_), and carboxyl (−COOH) (Figure S2). These groups can form strong hydrogen bonds with water, so PAMAA/CMC-Na hydrogels have high water content. In PAMAA/CMC-Na/Fe hydrogels, the coordination crosslinking between Fe^3+^ and the carboxyl groups resulted in the formation of hydrophobic structures in the polymer main chain. Thus, the hydrogen bond between P(AM-CO-AA)/CMC-Na/Fe^3+^ hydrogel and water (H_2_O) is weakened, and its water content is reduced.

As shown in Figure 2b,c, with the increase in n_AA_:n_AM_, the water content of the hydrogel gradually decreases from 80.53% to 74.49%. With the increase in CMC-Na concentration, the water content of the hydrogel gradually decreases from 82.43% to 77.35%. The increase in the n_AA_:n_AM_ ratio and CMC-Na concentration results in an increase in carboxyl (COO^−^) content, which enhances the coordination crosslinking between Fe^3+^ and the carboxyl groups, leading to a denser internal structure of the hydrogel. Therefore, the more hydrophobic structures formed in the polymer backbone weaken the hydrogen bonding interaction between water gel and water. As shown in Figure 2d,e, with the increase in Fe^3+^ concentration, the water content of the hydrogel gradually decreases from 80.64% to 76.68%. As the soaking time of Fe^3+^ solution increases, the water content of the hydrogel gradually decreases from 80.66% to 75.63%. The higher the Fe^3+^ content and the longer coordination crosslinking time, the stronger the coordination crosslinking between Fe^3+^ and the carboxyl groups, resulting in an increased density of crosslinking. Consequently, this leads to the formation of more hydrophobic structures within the polymer backbone, thereby weakening its hydrogen bonding with water (Figure S5).

### 3.2. Anti-Swelling Properties of PAMAA/CMC-Na/Fe Hydrogels

PAMAA hydrogel was synthesized using a free radical polymerization method as chemical cross-linking network, which was then soaked into 0.06 M FeCl_3_·6H_2_O aqueous solution to build the physical hydrogel. The formed dual-network hydrogels possess better performance in mechanical properties and swelling resistance. The reasons for the difference in anti-swelling properties of the dual-network hydrogels were studied.

**Figure 3:**
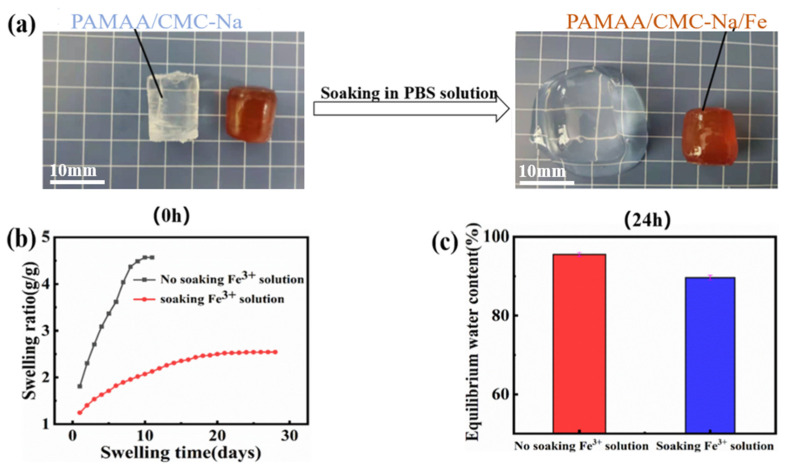
The stability and swelling of hydrogels after soaking in PBS solution (pH = 7.4) for 24 h: (**a**) physical state, (**b**) swelling ratio, and (**c**) equilibrium water content of PAMAA/CMC-Na and PAMAA/CMC-Na/Fe hydrogels.

Due to the existence of hydrophilic groups and macroporous network structure, most hydrogels swell when exposed to water, which greatly limits their practical applications. Observed in Figure 3a, the PAMAA/CMC-Na hydrogel is less stable and swelled, while the formed PAMAA/CMC-Na/Fe hydrogel basically keeps its original volume with high stability in PBS. We suggest that the hydrogels possess chemically cross-linked PAMAA network and physical dynamic cross-links of ionic coordination (Fe^3+^ and -COO^−^) network. In the meantime, the physical network of CMC-Na was formed owing to the movement and reorganization of its chains. Both factors improve the mechanical and anti-swelling properties of PAMAA/CMC-Na/Fe hydrogel. The thermal stability of the hydrogel was also improved. (Figure S3) The coordination crosslinking between Fe3+ and carboxyl group affects the hybrid orbitals of C and O, leading to the migration of their XPS peaks (Figure S4).

Figure 3b,c show the characteristic of the PAMAA/CMC-Na hydrogels without soaking in the Fe^3+^ solution, which reveal a more rapid increase in swelling rate as the duration of immersion increases. It reaches the swelling equilibrium in a short time (10 days) with a higher equilibrium swelling ratio (4.56 g/g) and a higher equilibrium water content (95.53%). In contrast, the hydrogels show a milder increase in swelling rate and a longer time to reach swelling equilibrium (28 days). In addition, it turns out to be a lower equilibrium swelling rate (2.52 g/g), and a lower equilibrium water content (89.61%). These results indicate that the PAMAA/CMC-Na/Fe hydrogels have better anti-swelling properties in the PBS solution than PAMAA/CMC-Na hydrogels.

**Figure 4:**
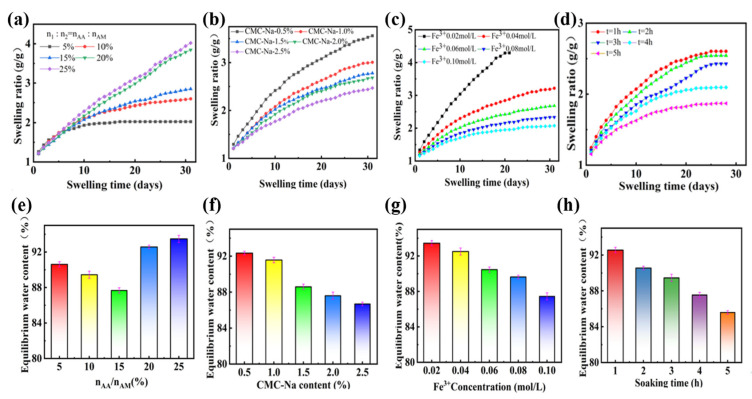
Effects of different n_AA_:n_AM_ on (**a**) swelling ratio and (**e**) equilibrium water content of the PAMAA/CMC-Na/Fe hydrogels. Effects of different CMC-Na concentrations on (**b**) swelling ratio and (**f**) equilibrium water content of the PAMAA/CMC-Na/Fe hydrogels. Effects of different Fe^3+^ concentrations on (**c**) swelling ratio and (**g**) equilibrium water content of the PAMAA/CMC-Na/Fe hydrogels. Effects of different soaking time on (**d**) swelling ratio and (**h**) equilibrium water content of the PAMAA/CMC-Na/Fe hydrogels.

The stability of PAMAA/CMC-Na/Fe hydrogels with different n_AA_:n_AM_ and CMC-Na concentration in PBS solution was further tested. As shown in Figure 4a–d, with the extension of the swelling time, the swelling ratio of the PAMAA/CMC-Na/Fe hydrogel generally climbed. As shown in Figure 4a,e, With the increase in n_AA_:n_AM_, the curve exhibits a higher swelling rate and ends up with a higher equilibrium swelling ratio. The equilibrium water content meets the minimum at 87.67% when n_AA_:n_AM_ is 15%. The difference of monomer proportion n_AA_:n_AM_ appears after 7 days of swelling times. In terms of the extension of soaking time, the swelling ratio of PAMAA/CMC-Na/Fe hydrogel gradually increased with the increase in swelling time (Figure 4b,f). As shown in Figure 4c,g, the equilibrium water content and the equilibrium swelling ratio gradually decrease with the increase in the CMC-Na concentration. By altering the duration of Fe^3+^ solution immersion, a gradual reduction in the swelling ratio of PAMAA/CMC-Na/Fe hydrogel was observed with increasing soaking time (Figure 4d,h).

### 3.3. pH-Dependent Anti-Swelling Property of PAMAA/CMC-Na/Fe Hydrogels

**Figure 5:**
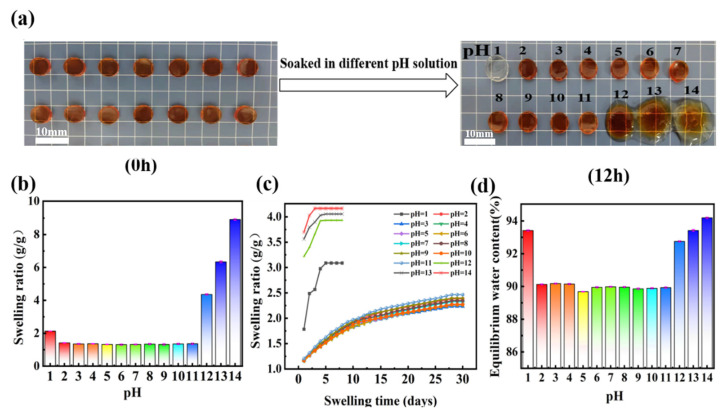
Properties of PAMAA/CMC-Na/Fe hydrogels after soaking in solution of different pH: (**a**) physical state, (**b**) swelling ratio (12 h), (**c**) swelling ratio (30 days), and (**d**) equilibrium water content.

The anti-swelling property of the created hydrogels under different pH was further explored. After soaking in the pH = 1 solution for 12 h, the color of PAMAA/CMC-Na/Fe hydrogel changed from yellow to transparent (Figure 5a). When the pH was adjusted to 2~11, the color and shape of the hydrogel remained unchanged after soaking for the same period. After soaking in the solutions (pH = 12, 13, and 14) for 12 h, the color and shape of hydrogels changed significantly. The main reason was that the carboxyl groups in the hydrogel are protonated by a large amount of H^+^, and then a large amount of Fe^3+^ is replaced in the solution in the hydrogel network due to the immersion in strongly acidic solution (pH = 1). Many hydrophilic groups in hydrogels, such as amino, acylamino, and carboxyl groups, establish robust hydrogen bonds with molecules of water. Due to the coordination and cross-linking between Fe^3+^ and the carboxyl group in CMC-Na and PAMAA many hydrophobic structures are formed in the main chain structure of the polymer, weakening the hydrogen bond with water molecules. Therefore, the immersed in pH = 2~11 solutions remained good structure. In terms of alkaline solution (pH ≥ 12), Fe^3+^ ions in the PAMAA/CMC-Na/Fe hydrogels were complexed by a large amount of OH^−^, reducing the interaction and bonding strength between Fe^3+^ and the carboxyl group. As a result, the anti-swelling property of the PAMAA/CMC-Na/Fe hydrogels became worse.

In addition, it indicated that the swelling rate of the hydrogel that immersed in pH = 1 solution for 12 h (swelling ratio of 2.13 g/g) was higher than that immersed in pH = 2~11 solutions for 12 h (swelling ratio of 1.31 g/g), as indicated in Figure 5b. The hydrogels immersed in pH = 12~14 solutions reached swelling ratios of 4.36~8.91 g/g. Meanwhile, when the pH was increased continuously, the swelling rates rise gradually.

The swelling behavior of the hydrogels can be affected by the soaking time. From Figure 5c it is evident that the swelling ratio of the hydrogels gradually increase with the extension of soaking time in different pH solutions. In the soaking solution (pH = 1), the swelling ratio of the PAMAA/CMC-Na/Fe hydrogel increases rapidly, and then it reaches the equilibrium swelling in a short time with a high equilibrium swelling ratio. In the soaking solution (pH = 2~11), the swelling ratio of the PAMAA/CMC-Na/Fe hydrogel slightly increases. It takes a long time to reach the equilibrium swelling and the equilibrium swelling ratio is low (about 2.23 g/g). However, in the soaking solutions (pH = 12~14), the swelling ratio of the PAMAA/CMC-Na/Fe hydrogel increases rapidly.

The effect of the solution pH on the equilibrium water content of hydrogels was studied. As depicted in Figure 5d, the hydrogel composed of PAMAA/CMC-Na/Fe exhibits a water content of 93.42% under immersion conditions (pH = 1). With the increase in the pH of the immersion solutions (pH = 2~11), the equilibrium water contents of the PAMAA/CMC-Na/Fe hydrogels are around 90%. With continuous increase in the pH to 12~14, the equilibrium water content of the PAMAA/CMC-Na/Fe hydrogels are increased to 92.75~94.19%. Therefore, we suggest that the solution pH could affect the equilibrium water content of hydrogels in our study.

### 3.4. Mechanical Performance of PAMAA/CMC-Na/Fe Hydrogels

**Figure 6:**
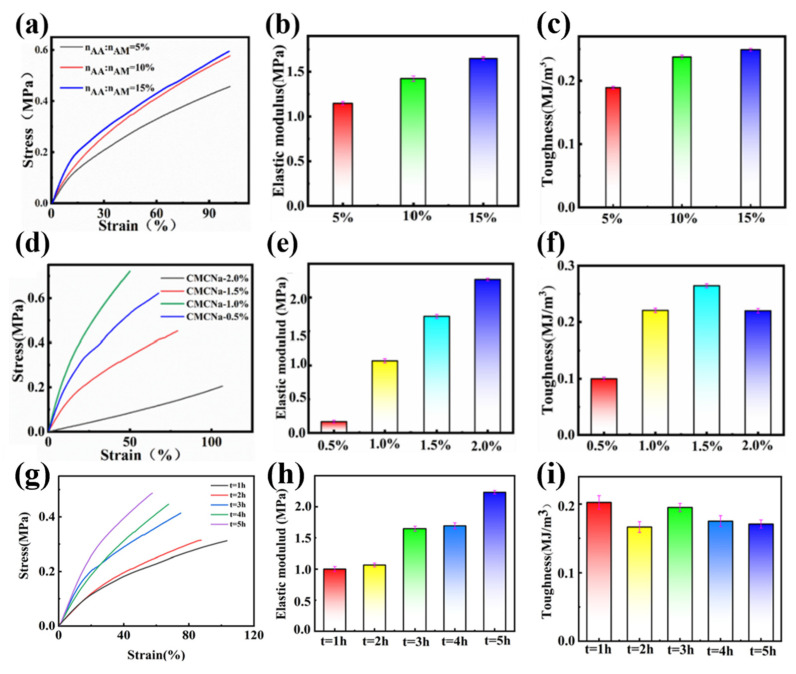
(**a**–**c**) The effects of different n_AA_:n_AM_ on (**a**) strain, (**b**) elastic modulus, and (**c**) toughness of the hydrogels. (**d**–**f**) The effects of different CMC-Na concentration on (**d**) strain, (**e**) elastic modulus, and (**f**) toughness of the hydrogels. (**g**–**i**) The effects of different soaking time on (**g**) strain, (**h**) elastic modulus, and (**i**) toughness of the hydrogels.

Different reaction rates were adjusted to generate different AA/AM ratios (n_AA_/n_AM_) of hydrogels and their properties were detected. The effects of different n_AA_/n_AM_ on the mechanical properties of the PAMAA/CMC-Na/Fe dual-network hydrogels were investigated by the tensile experiments. As presented in Figure 6a, the stress of the hydrogel gradually increases with the growth of strain. Meanwhile, its elastic modulus increases with the increase in the n_AA_:n_AM,_ from 1.1 to 1.6 MPa (Figure 6b), and its toughness increases from 0.18 to 0.24 MJ/m^3^ (Figure 6c).

We suggest the increased stress, elastic modulus, and toughness of hydrogels are ascribed to the increased amount of carboxyl group in CMC-Na and PAMAA with the increase in the n_AA_:n_AM_. In the hydrogel with higher n_AA_:n_AM_, the coordination and the interaction of Fe^3+^ and carboxyl groups are enhanced. The higher the cross-linking degree is, the denser physical cross-linking network forms. When hydrogels are stressed, the external energy can be dissipated through the breaking of chemical bonds, and other physical effects such as electrostatic interactions, hydrogen bonding, and coordination. Therefore, with the increase in the n_AA_:n_AM_, the mechanical properties can be enhanced significantly. 

The effects of the CMC-Na concentration on the strain, elastic, modulus, and toughness of hydrogels were further studied. Figure 6d illustrates the influence of different CMC-Na concentration on the mechanical characteristics of the dual-network hydrogels. The hydrogel’s stress is enhanced by manipulating the concentration of CMC-Na. Simultaneously, as the concentration of CMC-Na rises, the elastic modulus of the PAMAA/CMC-Na/Fe hydrogel gradually increases from 0.16 to 2.26 MPa (Figure 6e). In addition, the toughness of the PAMAA/CMC-Na/Fe hydrogel is the highest (0.26 MJ/m^3^) when the CMC-Na concentration is 1.5%, as shown in Figure 6f.

As the concentration of CMC-Na is elevated, there is an increase in the quantity of carboxyl groups present in the hydrogels. This leads to a promotion of both coordination and cross-linking between Fe^3+^ and the carboxyl groups in CMC-Na and PAMAA, leading to the creation of additional networks through physical cross-linking. Therefore, the strength of hydrogels is increased through mechanical means. However, when the carboxyl amount is too high, the cross-linking network is too dense, which will decrease the toughness of the hydrogel and make the hydrogel more brittle.

As shown in Figure 6g, the stress of the hydrogel gradually increases with the growth of strain. The elastic modulus of hydrogel increases gradually with the increase in soaking time of Fe^3+^ solution from 1 to 2.23 MPa (Figure 6h). Toughness firstly decreased from 0.1 to 0.08 MJ/m^3^, then increased to 0.09 MJ/m^3^ and decreased back to 0.085 MJ/m^3^ (Figure 6i).

The main reason is that as the Fe^3+^ solution immersion time increases, the more full the cross-linking effect, the more dense the cross-linking degree, more physical cross-linking network is formed, and the better the mechanical strength of the hydrogel can be maintained. (Figure S1) However, Fe^3+^ solution immersion time is too long, the formation of the network structure is too dense, the hydrogel toughness decreased, brittleness increased. When the hydrogel is in the stress–strain state, the P(AM-*co*-AA)/CMC-Na/Fe^3+^ hydrogel dissipates external energy by breaking chemical bonds. They can also dissipate external energy through physical effects such as electrostatic interactions, hydrogen bonding, and coordination crosslinking. Thus, the internal structure of the hydrogel is maintained, and the toughness and elasticity of the hydrogel are maintained.

Based on our research findings, immersing the material in a Fe^3+^ solution with a concentration range of 0.02–0.03 M for a duration of two hours demonstrates favorable mechanical properties. However, it is important to note that higher concentrations and prolonged soaking times may lead to brittleness in the material. 

### 3.5. Anti-Freezing Properties of Hydrogels

**Figure 7:**
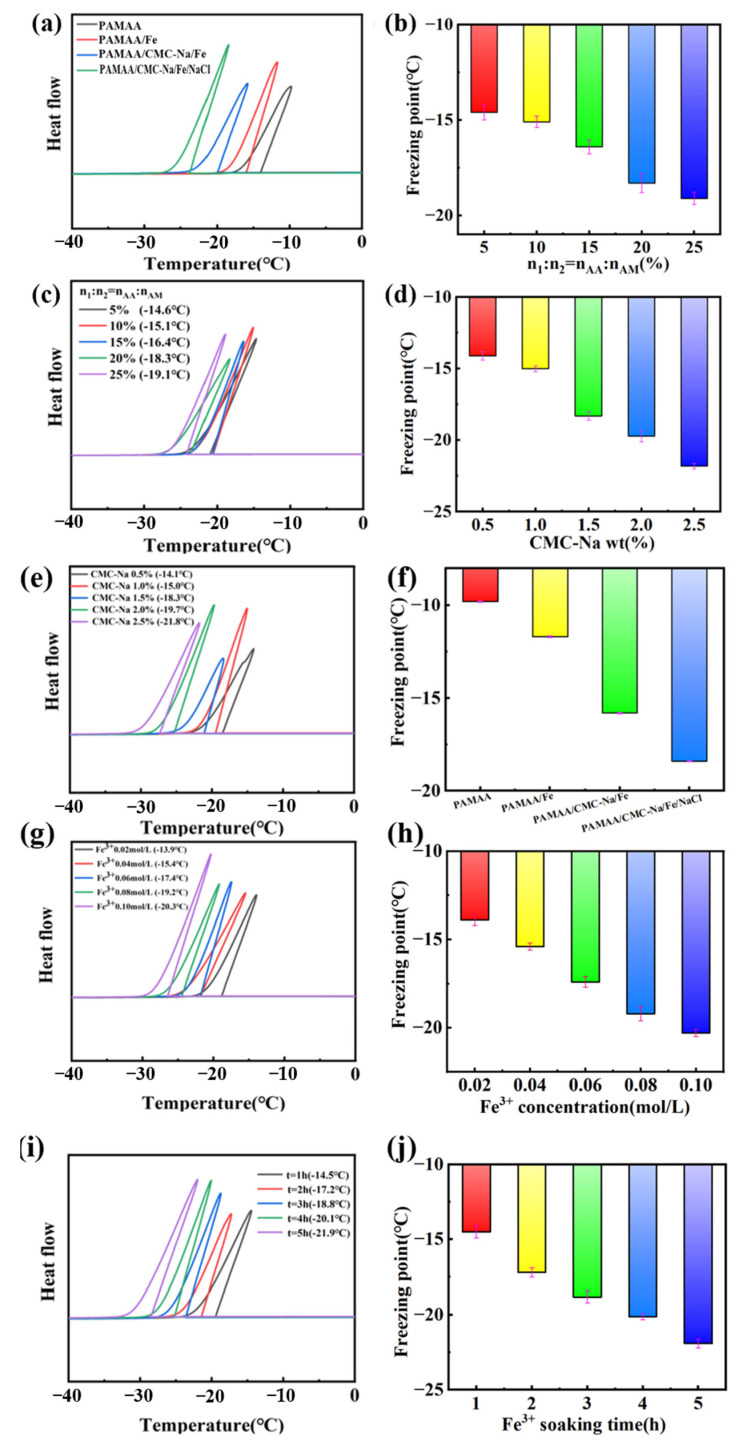
(**a**) DSC curves of hydrogels. Effects of (**c**) different n_AA_:n_AM_ and (**e**) CMC-Na concentration and (**g**) Fe^3+^ concentration and (**i**) Fe^3+^ soaking time on anti-freezing performance of the hydrogels. (**b**) Freezing point, and the effects of (**d**) different n_AA_:n_AM_ and (**f**) CMC-Na concentration and (**h**) Fe^3+^ concentration and (**j**) Fe^3+^ soaking time on the freezing point.

The anti-freezing properties of the created dual-network hydrogels was also investigated. As shown in Figure 7a, the exothermic peaks of the DSC curves of the PAMAA, PAMAA/Fe^3+^, and PAMAA/CMC-Na/Fe hydrogels and PAMAA/CMC-Na/Fe hydrogels reveal steadily offset toward the lower temperature. It can be found from Figure 7b that the freezing points of all three types of hydrogels constantly drop, indicating that the anti-freezing resistance gradually improves.

Figure 7c presents the exothermic peaks of the DSC curves of the PAMAA/CMC-Na/Fe hydrogels, which gradually shifts toward the lower temperature with the increase in the n_AA_:n_AM_. This indicates that the anti-freezing performance steadily improves with the increase in the n_AA_:n_AM_. Correspondingly, the freezing points shown in Figure 7e gradually decreases from −14.6 to −19.1 °C with the increase in the n_AA_:n_AM_.

In addition, with the rise in the CMC-Na concentration, the exothermic peaks gradually shift to the lower temperature direction in the DSC curves of the PAMAA/CMC-Na/Fe hydrogels, in shown in Figure 7e. We suggest that the anti-freezing performance of the dual-network hydrogels gradually improve with the increasing the CMC-Na concentration. It can also be found that from Figure 7f the freezing points of the hydrogels gradually decrease from −14.1 to −21.8 °C with the rise in the CMC-Na concentration.

As shown in Figure 7g, with the increase in Fe^3+^ concentration, the exothermic peak of P(AM-*co*-AA)/CMC-Na/Fe^3+^ hydrogel DSC curve gradually shifted to the lower temperature. This indicates that the frost resistance gradually improved. As shown in Figure 7h, the freezing point of P(AM-*co*-AA)/CMC-Na/Fe^3+^ hydrogel gradually decreases from −13.9 °C to −20.3 °C with the increase in Fe^3+^ immersion concentration.

As shown in Figure 7i, with the improvement of Fe^3+^ solution immersing time, P (AM-*co*-AA)/CMC-Na/Fe^3+^ water gel DSC curve, the exothermic peak gradually shifts to the direction of lower temperature, which shows that the performance of antifreeze improves gradually. As shown in Figure 7j, the freezing point of P(AM-*co*-AA)/CMC-Na/Fe^3+^ hydrogel from −14.5 to −21.9 °C) gradually decreases with the increase in soaking time of the Fe^3+^ solution.

**Figure 8:**
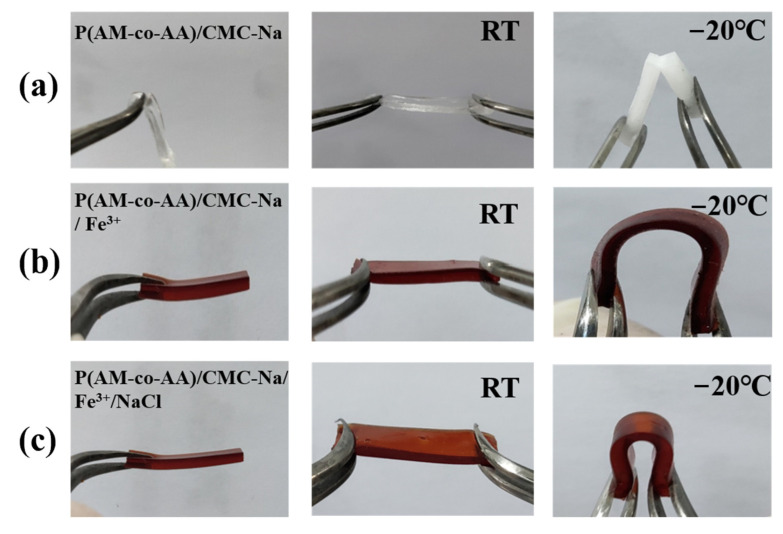
The flexibility performance of (**a**) P(AM-co-AA)/CMC-Na hydrogels, (**b**) P(AM-co-AA)/CMC-Na/Fe^3+^ hydrogels, and (**c**) P(AM-co-AA)/CMC-Na/Fe^3+^/NaCl hydrogels at room temperature and −20 °C.

According to Figure 8, the performance of the sample was evaluated after subjecting it to freezing treatment at −20 °C. At room temperature, PAMAA/CMC-Na hydrogels, PAMAA/CMC-Na/Fe^3+^ hydrogels, and PAMAA/CMC-Na/Fe^3+^/NaCl hydrogels exhibited stretchability. However, when exposed to −20 °C, the color of PAMAA/CMC-Na hydrogels turned milky and they fractured upon bending. In contrast, both PAMAA/CMC-Na/Fe^3+^ hydrogels and PAMAA/CMC-Na/Fe^3+^/NaCl hydrogels demonstrated excellent flexibility as they remained intact even after bending under such low temperatures.

Here are the reasons for the better anti-freezing performance through increasing the n_AA_:n_AM_, CMC-Na concentration, Fe^3+^ concentration, and Fe^3+^ soaking time. The high water-content of the PAMAA hydrogels and PAMAA/CMC-Na hydrogels provide more hydrophilic groups such as hydroxy, amino, and carboxyl groups. When the external ambient temperature is lower than 0 °C, the water molecules are easy to gather and freeze, which decreases the anti-freezing performance of hydrogels. Firstly, as the n_AA_:n_AM_, CMC-Na concentration, Fe^3+^ concentration, and Fe^3+^ soaking time increased, coordination crosslinking between CMC-Na and PAMAA was enhanced. As a result, a large number of hydrophobic structures are formed in the polymer backbone structure, weakening the hydrogen bond between the hydrogel and water. The nucleation and growth of ice crystals are significantly inhibited, leading to a significant reduction in the freezing point (freezing temperature) of hydrogels. Secondly, the presence of ions had a significant impact in enhancing the ability to withstand freezing temperatures by decreasing water molecule aggregation and reducing internal hydrogen bonding. The incorporation of NaCl induces a robust electrostatic effect, effectively impeding water accumulation. As a result, the hydrogel was able to retain both mechanical flexibility and ionic mobility, even under conditions of reduced temperature. This leads to significant attenuation in both nucleation and growth of ice crystals, resulting in a substantial reduction in the freezing point (freezing temperature) of hydrogels. Consequently, PAMAA/CMC-Na/Fe^3+^ hydrogels and PAMAA/CMC-Na/Fe^3+^/NaCl hydrogels exhibit remarkable flexibility even under low temperatures. Therefore, our designed dual-network hydrogels can be potentially assembled as a wearable, anti-freezing, and sensitive sensor for utilization in challenging conditions.

### 3.6. Conductivity of the PAMAA/CMC-Na/Fe Hydrogels

The migrating conductive ions helps weakening hydrogen bonding and increasing the conductivity of materials. To investigate the conductivity of the designed dual-network hydrogels, the prepared hydrogels were cut into 6 mm radius and 2 mm thickness cylindrical samples and the stable voltage was tested.

**Figure 9:**
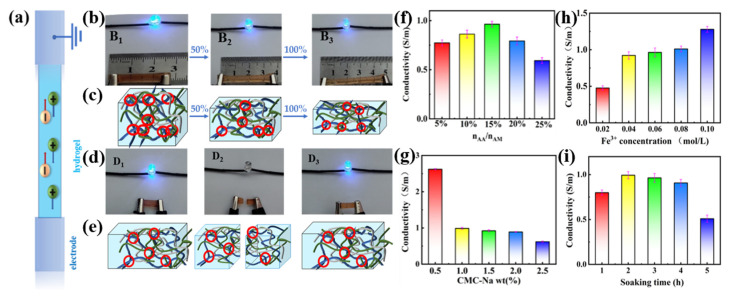
Conductivity testes of PAMAA/CMC-Na/Fe hydrogels: (**a**) Simplified depiction of the movement of ions within the hydrogels. (**b**) The bulb brightness and (**c**) schematic drawing of the hydrogels under different strains: (**B_1_**) 0, (**B_2_**) 50%, and (**B_3_**) 100%. (**d**) The bulb brightness and (**e**) schematic drawing of hydrogels under different states: (**D_1_**) initial, (**D_2_**) cutting, (**D_3_**) electrical healing. Effect of different (**f**) n_AA_:n_AM_ and (**g**) CMC-Na concentration and (**h**) Fe^3+^ concentration and (**i**) Fe^3+^ soaking time on the conductivity of hydrogels.

Simplified depiction of the movement of ions within the PAMAA/CMC-Na/Fe hydrogel is shown in Figure 9a. PAMAA/CMC-Na/Fe hydrogels contain a large number of freely migrating conductive ions such as Fe^3+^, Cl^−^, and Na^+^. Under the action of electric field, the free Fe^3+^ migrates to the cathode and Cl^−^ migrates to the anode. Therefore, PAMAA/CMC-Na/Fe hydrogel can be used as a wire to form a closed loop with the LED bulb. It can be found that the PAMAA/CMC-Na/Fe hydrogel is connected to the LED bulb as a wire to form a complete circuit (Figure 9b). The brightness of the bulb weakens with hydrogel under different strains: (B_1_) 0, (B_2_) 50%, and (B_3_) 100%. Schematic illustration of the PAMAA/CMC-Na/Fe hydrogel at different strains is shown in Figure 9c. As the tensile strain increases, the migration of free ions in the hydrogel is blocked. This, in turn, leads to the decrease in the brightness of the LED bulb.

The hydrogel was then placed as a wiring circuit, such as the tests of (D_1_) initial, (D_2_) cutting, and (D_3_) electrical healing (Figure 9d). It can be found that the LED bulb goes off when the PAMAA/CMC-Na/Fe hydrogel-like strip is cut off. However, the LED bulb lights up again after the two cut hydrogel sections are reattached together, and the brightness remained the same as the initial brightness, which indicates that hydrogel has conductive and self-healing properties. Figure 9e schematically shows the PAMAA/CMC-Na/Fe hydrogel in initial, cutting, and electrical recovery states. The hydrogel contains many freely migrating conductive ions such as Fe^3+^, Cl^−^, and Na^+^. More conductive ions can be contained in the polymer backbone. Thus, the content of freely migrating conductive ions increases, leading to the recovery in conductivity.

Through the statistical analysis, the conductivity of the PAMAA/CMC-Na/Fe hydrogel reaches top at 0.96 S/m with the increase in n_AA_:n_AM_ (Figure 9f), which is mainly due to the increase in the n_AA_:n_AM_, the increase in carboxyl group (-COO^−^) content, and the adequate coordination between Fe^3+^ and the carboxyl groups. More Fe^3+^ can be included in the polymer backbone, which results in more freely migrating conductive ions, leading to an increase in conductivity. When the n_AA_:n_AM_ is too high, the amount of carboxyl groups is too large. The cross-linking of Fe^3+^ with carboxyl groups is enhanced, and the cross-linking degree is much higher.

As depicted in Figure 9g, the conductivity of the PAMAA/CMC-Na/Fe hydrogel gradually decreases from 2.62 to 0.6 S/m as the CMC-Na concentration rises. It is mainly due to the increase in the carboxyl group content with the rise in the CMC-Na concentration. The cross-linking of Fe^3+^ with the carboxyl group is enhanced, and the cross-linking network becomes denser. Thus, the free migration of conducting ions is restricted, resulting in decreased conductivity. Therefore, the conductivity gradually decreases with the increase in the CMC-Na concentration.

As depicted in Figure 9h, the conductivity of PAMAA/CMC-Na/Fe hydrogel gradually increases from 0.47 S/m to 1.27 S/m as the concentration of Fe^3+^ increases. This is mainly due to the increase in the concentration of Fe^3+^, which causes an enhancement in the coordination cross-linking between CMC-Na and PAMAA. As a result, more Fe^3+^ can be contained in the main chain of polymerization, leading to more freely migrating conductive ions and thus lower resistance values and higher conductivity. The increase in the concentration of Fe^3+^ ions results in an enhanced conductivity in the PAMAA/CMC-Na/Fe hydrogel. By comprehensive comparison, it reveals that the hydrogel immersed in a 0.06 M Fe^3+^ solution exhibits both a lower freezing point and enhanced conductivity.

As shown in Figure 9i, the conductivity of P(AM-*co*-AA)/CMC-Na/Fe^3+^ hydrogel firstly increased from 0.79 S/m to 0.96 S/m, then decreased to 0.5 S/m with the increase in Fe^3+^ soaking time. Mainly attributed to the prolonged soaking duration, the coordination crosslinking between Fe^3+^ and the carboxyl group becomes more comprehensive, facilitating a greater incorporation of Fe^3+^ into the polymerization main chain. Consequently, there is an increase in the content of freely migrating conductive ions, leading to a reduction in resistance value and an initial rise in conductivity. However, excessive soaking time of Fe^3+^ solution intensifies the coordination crosslinking between Fe^3+^ and the carboxyl group, resulting in a denser degree of crosslinking. This restricts the free migration of conducting ions, causing an elevation in their resistance value and a decline in their conductivity.

## 4. Conclusions

In summary, PAMAA/CMC-Na/Fe hydrogels with dual-networks were fabricated by socking the as-prepared PAMAA/CMC-Na hydrogels into FeCl_3_ solution. In this case, the initial chemical cross-linking network consisted of PAMAA. Fe^3+^ and carboxyl (in CMC-Na and PAMAA) were coordinated and cross-linked to obtain the second network via physical cross-linking. The effects of different n_AA_/n_AM_ and CMC-Na concentrations on the anti-swelling, anti-freezing, and mechanical properties of the dual-network hydrogels were investigated. The obtained results proved that the dual-network hydrogels revealed good swelling resistance and frost resistance. Due to the synergistic effect caused by chemical and physical cross-linking networks, the dual-network hydrogels exhibited good swelling resistance and equilibrium swelling rate. The lowest freezing point was −21.8 °C, indicating good anti-freezing ability. In addition, the coordination between Fe^3+^ and the carboxyl may potentially reduce the strength of hydrogen bonding among water molecules, thereby impeding the formation and enlargement of ice crystals within hydrogels. With the increase in the n_AA_:n_AM_, the conductivity of the PAMAA/CMC-Na/Fe hydrogels reached to 0.96 S/m. As the concentration of CMC-Na rises, the conductivity of the hydrogel gradually decreased from 2.62 to 0.62 S/m. It is expected that the dual-network hydrogels with high mechanical properties, anti-swelling properties, anti-freezing ability, self-healing, and electrical conductivity can adapt to a wide range of pH environments and broaden their applications in various fields.

## Figures and Tables

**Table 1:** The compositions of hydrogels prepared in this work.

m_AA_/n_AM_ (wt%)	CMC-Na (wt%)	APS (wt%)	TEMED (wt%)	Fe^3+^ (wt%)	Soaking Time (h)
15	1.5	1	2	/	/
5	1.5	1	2	0.06	3
10	1.5	1	2	0.06	3
15	1.5	1	2	0.06	3
20	1.5	1	2	0.06	3
25	1.5	1	2	0.06	3
15	0.5	1	2	0.06	3
15	1	1	2	0.06	3
15	1.5	1	2	0.06	3
15	2	1	2	0.06	3
15	2.5	1	2	0.06	3
15	1.5	1	2	0.02	3
15	1.5	1	2	0.04	3
15	1.5	1	2	0.06	3
15	1.5	1	2	0.08	3
15	1.5	1	2	0.1	3
15	1.5	1	2	0.06	1
15	1.5	1	2	0.06	2
15	1.5	1	2	0.06	3
15	1.5	1	2	0.06	4
15	1.5	1	2	0.06	5

## Data Availability

Data are contained within the article.

## Supplementary Materials

The following supporting information can be downloaded at: https://www.mdpi.com/article/10.3390/ma17061275/s1, Figure S1: SEM images of hydrogels of different components; Figure S2: FTIR spectra of different hydrogels; Figure S3: DSC curves of different hydrogels; Figure S4: XPS of scan curves for different samples; Figure S5: Water content of hydrogel under different conditions.

## Abbreviations

CMC-Na	sodium carboxymethylcellulose
P(AM-*co*-AA)	Poly (acrylamide-*co*-acrylic acid)
Fe^3+^	ferric iron
AM	Acrylamide
AA	acrylic acid
n_AA_:n_AM_	Molar ratios of AA/AM
NaCl	sodium chloride
FeCl_3_	ferric trichloride
MBAA	*N,N′*-methylenebisacrylamide
APS	ammonium persulfate
PBS	phosphate buffered saline
TEMED	*N,N,N′,N′-*Tetramethylethylenediamine
DSC	Differential Scanning Calorimetry
SEM	Scanning Electron Microscopy
FTIR	Fourier Transform Infrared Spectroscopy
XPS	X-ray photoelectron spectrometer
LED	Light Emitting Diode
DN	double network
PAM	polyacrylamide
PAA	polyacrylic acid
