# Supplementary material for: Facile Synthesis of Dual-Network Polymer Hydrogels with Anti-Freezing, Highly Conductive, and Self-Healing Properties
Source: Materials (Basel). 2024 Mar 10;17(6):1275. doi: 10.3390/ma17061275 (PMC10972050; doi:10.3390/ma17061275)
Supplement: Supplementary file 1 [file materials-17-01275-s001.zip › materials-2887346-supplementary.pdf]

Supporting Information

Swelling-resistant, Anti-Freezing and Conductive Hydrogels with dual-crosslinked networks

Yuchen Jin[1], Lizhu Zhao[1], Ya Jiang[1], Xiaoyuan Zhang*[,1], Zhiqiang Su*[,1]

1. State Key Laboratory of Chemical Resource Engineering, Beijing Key Laboratory of Advanced Functional Polymer Composites, Beijing University of Chemical Technology, Beijing 100029, China.

* Corresponding authors. E-mail addresses: suzq@mail.buct.edu.cn (Z.S.), Xiaoyuan.Zhang@buct.edu.cn (X.Z.)

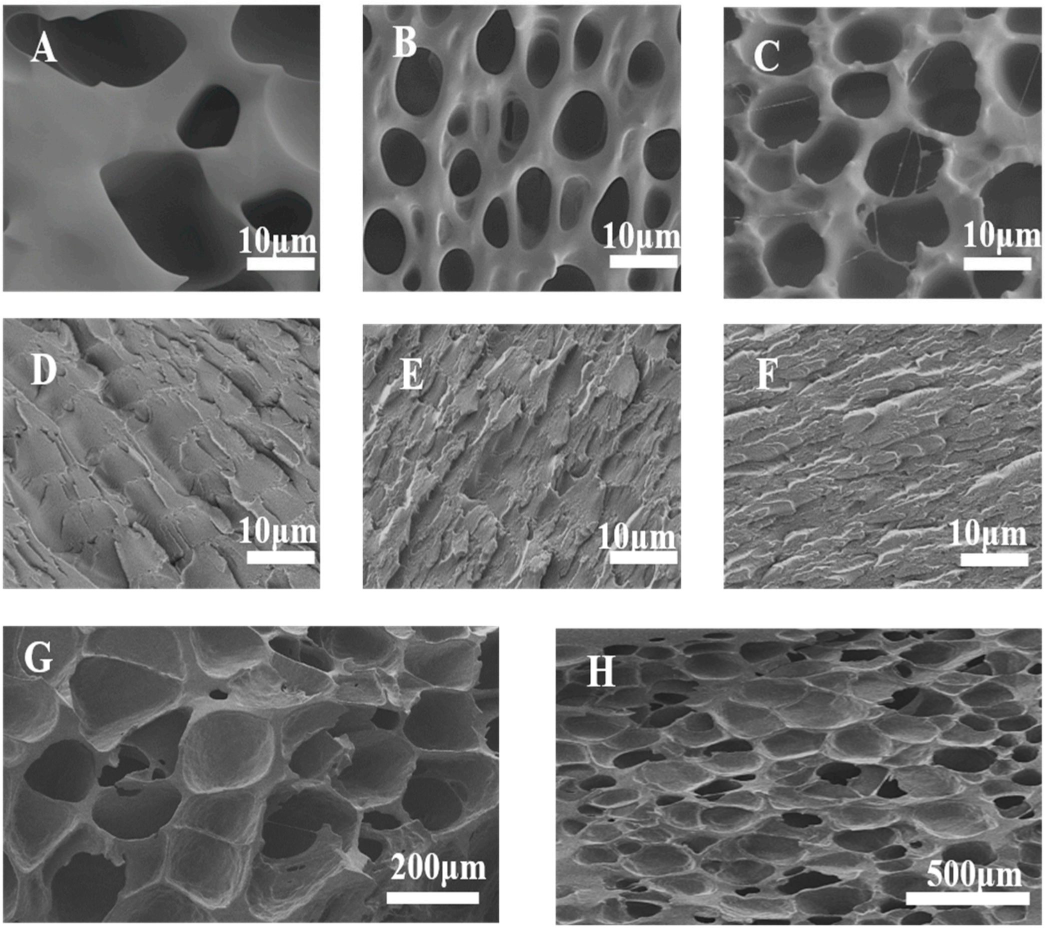

Figure S1. SEM images of the PAM hydrogel (A), the P(AM-co-AA) hydrogel (B) , the P(AM-co-AA)/CMC-Na hydrogel (C), the P(AM-co-AA)/Fe^{3+} hydrogel (D), the P(AM-co-AA)/CMC-Na/Fe^{3+} hydrogel (E), P(AM-co-AA)/CMC-Na/Fe^{3+} /NaCl hydrogel (F) , P(AM-co-AA)/CMC-Na/Fe^{3+}(G,200μm) and (H,500μm).

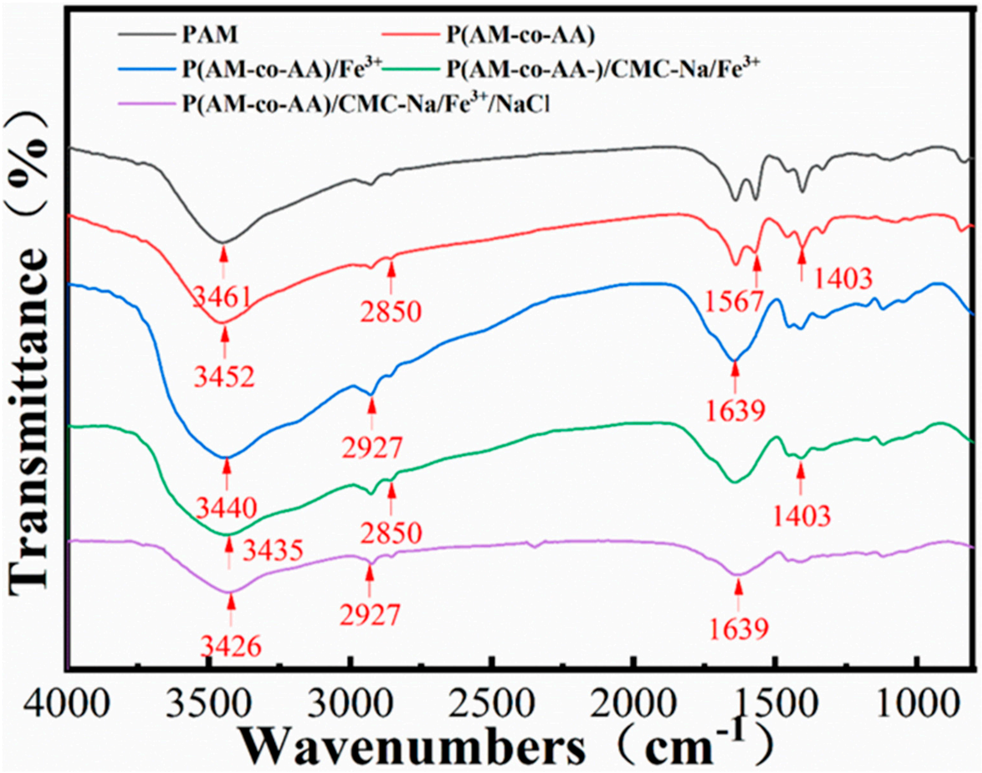

Figure S2. FTIR spectra of different hydrogels

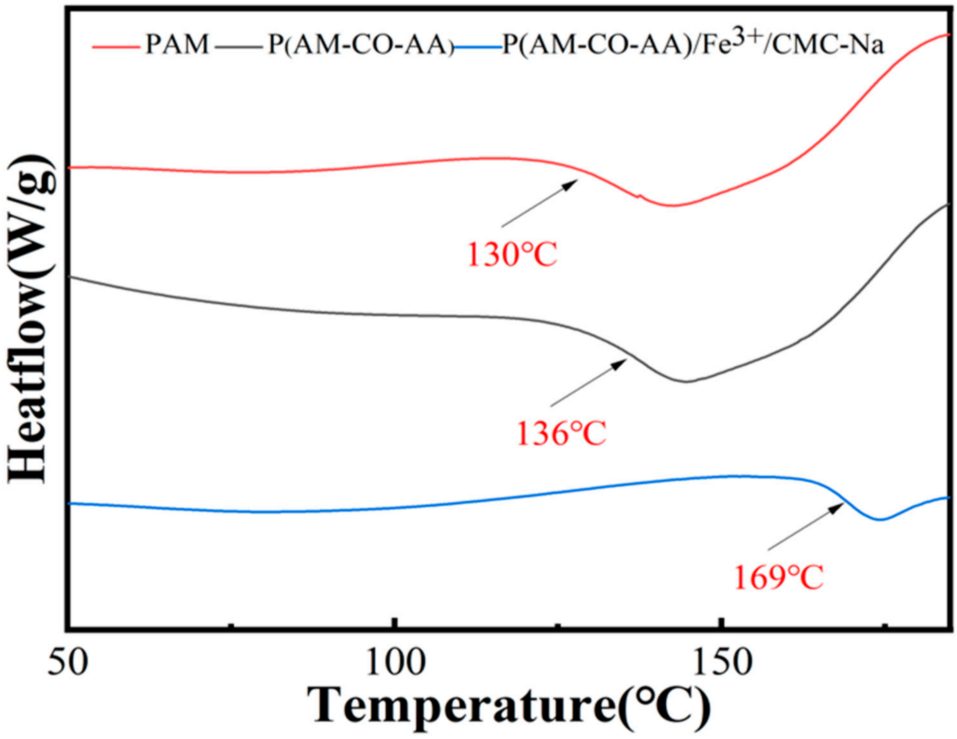

Figure S3. DSC curves of PAM, P(AM-co-AA), and P(AM-co-AA)/ CMC-Na / Fe^{3+}

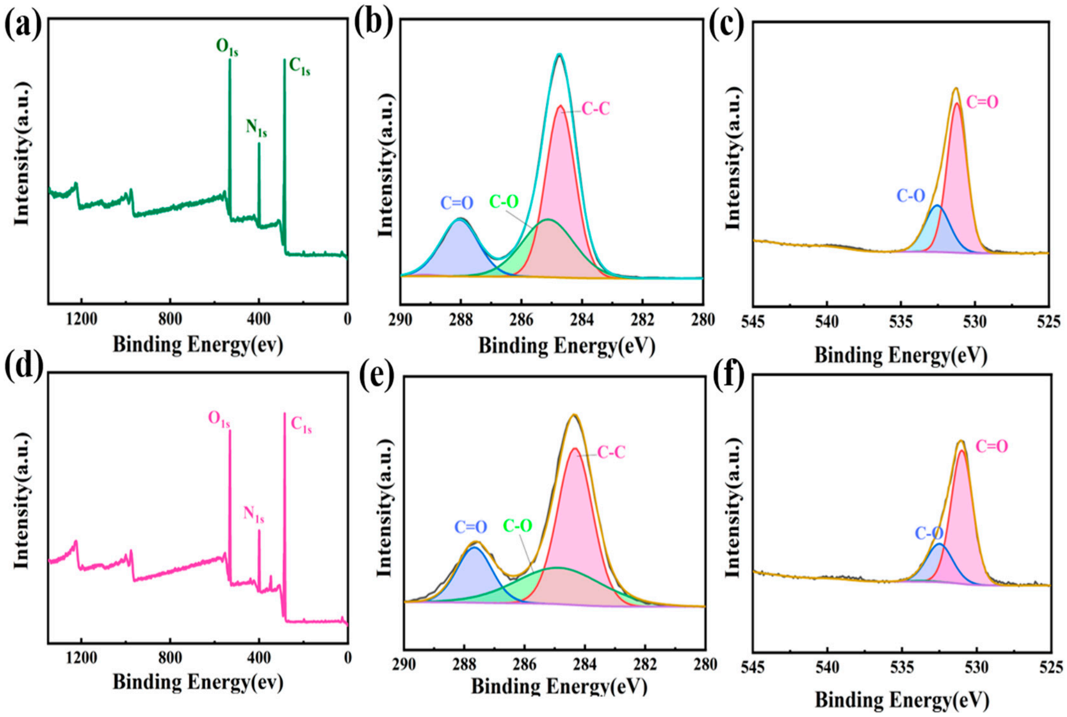

Figure S4. (a) XPS of the P(AM-co-AA)/ CMC-Na wide sweep curve,(b) XPS of the P(AM-co-AA)/ CMC-Na C_{1s} narrow sweep curve,(c) XPS of the P(AM-co-AA)/ CMC-Na O_{1s} narrow sweep curve,(d) XPS of the P(AM-co-AA)/ CMC-Na / Fe^{3+} wide sweep curve,(e) XPS of the P(AM-co-AA)/ CMC-Na / Fe^{3+} C_{1s} narrow sweep curve,(f) XPS of the P(AM-co-AA)/ CMC-Na / Fe^{3+} O_{1s} narrow sweep curve.

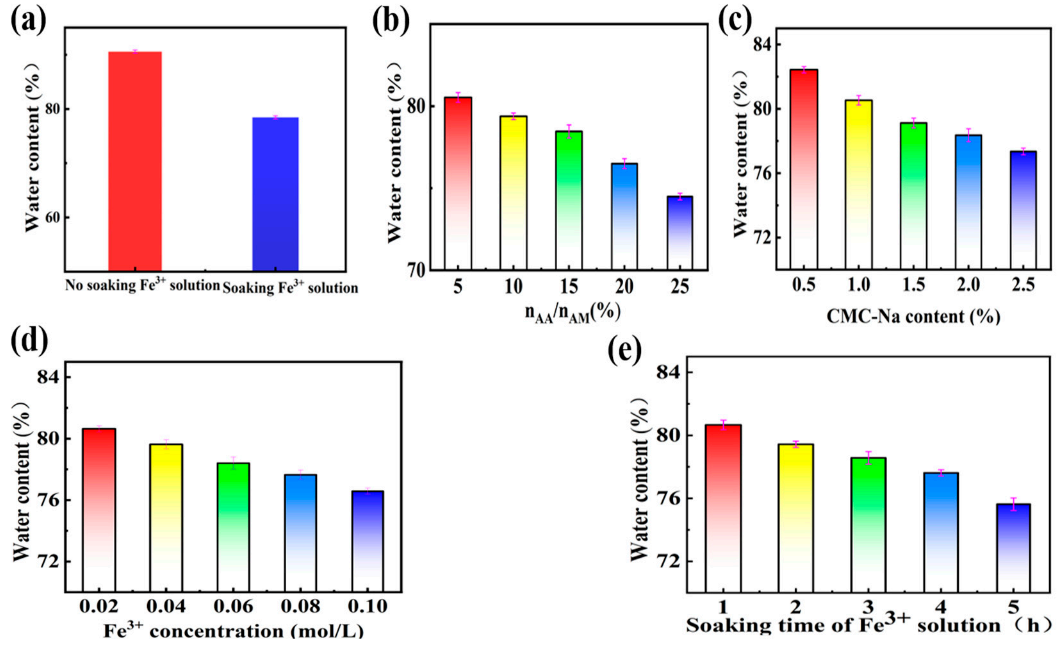

Figure S5. (a) Water content of P(AM-co-AA)/ CMC-Na hydrogels and P(AM-co-AA)/ CMC-Na/Fe^{3+} hydrogels,(b,c) Effects of different CMC-Na concentration and

n_{AA}:n_{AM} on Water content of P(AM-co-AA)/ CMC-Na/Fe^{3+} hydrogels. (d,e) Effects of soaking different Fe^{3+} concentration and soaking different Fe^{3+}solution time on Water content of P(AM-co-AA)/ CMC-Na/Fe^{3+} hydrogels.
